# School Climate, Moral Disengagement and, Empathy as Predictors of Bullying in Adolescents

**DOI:** 10.3389/fpsyg.2021.656775

**Published:** 2021-05-04

**Authors:** Carlos Montero-Carretero, Diego Pastor, Francisco Javier Santos-Rosa, Eduardo Cervelló

**Affiliations:** ^1^Sports Research Centre (Department of Sport Sciences), University Miguel Hernández of Elche, Elche, Spain; ^2^Department of Sports and Computer Science, University Pablo de Olavide, Sevilla, Spain

**Keywords:** school climate, moral disengagement, empathy, bullying, adolescence

## Abstract

Our work aimed to study the relationships between different dimensions of school climate, moral disengagement, empathy, and bullying behaviors (perpetration and victimization). The study sample consisted of 629 students (304 boys and 325 girls) aged 12–14 years (*M* = 12.55, *SD* = 0.67). Results showed how different dimensions of school climate predicted moral disengagement, empathy, and victimization, and these, in turn, predicted bullying perpetration. The results show the need to generate favorable educational environments to reduce the levels of moral disengagement and victimization and to increase empathy in students as a strategy to prevent negative consequences related to bullying.

## Introduction

Bullying is one of the main coexistence problems in schools worldwide (Chan and Wong, 2015a). It is a form of violence among young people with specific characteristics (Gladden et al., 2014). According to these authors, bullying has been defined as aggressive behavior that is repeated over time, with the intention of causing physical, psychological, social, or educational harm, where there is an imbalance of power between aggressors and victims, who are not siblings or current dating partners. Depending on the type of aggression, four subtypes of bullying can be differentiated: physical, verbal, relational, and cyber (Stubbs-Richardson et al., 2018).

Serious consequences are caused for all social agents involved (aggressors, victims, and onlookers). Usually, bully-victims suffer the most harm, experiencing anxiety, depression, absenteeism, poor academic performance (Gini and Pozzoli, 2009; Wolke and Lereya, 2015; Chu et al., 2019; Espelage and Hong, 2019), eating disorders, low self-esteem, loneliness, poor quality of relationships, self-harm, and suicidal thoughts which sometimes materialize (Van Geel et al., 2014; Estévez et al., 2019; Peng et al., 2020).

World prevalence data, including those of Spain (Chan and Wong, 2015b, 2020; Zych et al., 2017a; Inchley et al., 2020; Arhuis-Inca et al., 2021), justify the concern of the scientific and educational community to make further progress in the study of this complex social phenomenon to eradicate it. For this purpose, one of the main concerns has been and continues to be an in-depth understanding of the causes that lead young people to perpetrate bullying.

In line with social-ecological theory, many works have pointed out that bullying is the product of an interaction between individual characteristics and different layers of social contexts (Hong and Espelage, 2012; Romera et al., 2020). Thus, school contexts have been highly analyzed concerning bullying. The study of the context in learning environments comes from afar when Lewin (1936) prompted the study of psychology to go from focusing on the individual to focusing on the process among individuals. In this line, the study of the school climate has been gaining prominence among researchers concerned about bullying.

Although different individual characteristics affect bullying, moral disengagement and empathy have shown their predictive character for bullying perpetration (Gini et al., 2014). It also seems that past experiences can be decisive, and young people who intimidate others have often been victims in the past (Cook et al., 2010; Chan and Wong, 2015a, 2020; Zych et al., 2017b). Despite the broad body of knowledge about bullying precursors, we do not know whether a model has been previously tested that analyzes the moderating effect of the different psychosocial dimensions of the school climate on moral disengagement, empathy, and victimization as precursors of bullying perpetration.

### School Climate and Bullying

School climate has been defined as “the quality and character of school life” which includes “rules, values, and expectations that help people feel socially, emotionally, and physically safe” (Cohen et al., 2009, p. 182). A review focused on the multidimensional nature of school climate (Lewno-Dumdie et al., 2019) reveals the existence of 18 measuring instruments generated between 1975 and 2017, reflecting the great interest that its study has awakened and still awakens (Alonso-Tapia et al., 2020).

Despite the possibilities offered by the comprehension of the construct, a positive climate is determined by rules, goals, ideals, interpersonal relationships, instructional practices, and organizational structures within a school, which achieve an environment of respect, support for individuals, high quality of social relations, positive emotional environments, and physical safety (Appleton et al., 2008; Cohen et al., 2009; Thapa et al., 2013). Some of the most studied dimensions of school climate have been: the support that students perceive from their teachers, the clarity of the rules concerning bullying in schools, the communication channels enabled for students to report their problems, the student's perception of the acceptance of diversity within the people who live together in the schools, and the quality of the relationships between the students and their feeling of belonging to the school (Aldridge et al., 2018).

Positive school climate has been associated with many adaptive consequences such as students' self-esteem, self-concept, physical health, mental health, effort, and academic achievement (Cohen et al., 2009; Jamal et al., 2013; Thapa et al., 2013; Wang and Degol, 2016). School climate has also been shown to be an important predictor of emotional and behavioral consequences (Wang et al., 2010). Changes in the school climate related to increases in discipline and order, as well as in the quality of the relationships between students and teachers, have been shown to be effective in reducing behavioral problems by helping to increase safety in school (Johnson and Templeton, 1999; Wang et al., 2010).

In this line, the negative relationships between positive school climate and the prevalence of bullying are well documented (e.g., Cook et al., 2010; Konishi et al., 2017). Thus, some characteristics of school climate such as supportive peer-peer (Demaray and Malecki, 2003; Li et al., 2011; Turner et al., 2014) and student-teacher relationships (Olweus, 1994; Demaray and Malecki, 2003; Li et al., 2011), connectedness and commitment to the school (Li et al., 2011; Turner et al., 2014), sense of belonging in school (Chan and Wong, 2019), clear limits and consequences for unacceptable behavior (Olweus, 1994), and normative beliefs concerning bullying in the entire school (Gendron et al., 2011) have been related to a decrease in bullying.

As some studies have shown, the most effective interventions to prevent bullying were based on developing some of the school climate's dimensions, such as peer relationships, teacher support, or tolerance and respect for diversity (Gaffney et al., 2019). Zych et al. (2017a) emphasized the relevance of generating peaceful climates in schools, involving all school social agents (Chan and Wong, 2015b).

In a study of more than 6,000 high school students in Australia (Aldridge et al., 2018), school climate predicted victimization through five of the six dimensions that were measured. It should be noted that the dimensions of school connectedness, rule clarity, and support of the teachers were negative predictors of victimization, whereas affirming diversity and reporting and seeking help positively predicted victimization. The authors justify finding these positive relationships considering that there might be school normative beliefs about diversity, making students who had a different conception of diversity and tolerance for diversity feel victimized and helpless when reporting information and asking for help. A recent study confirms the importance of encouraging these dimensions of the school climate to prevent bullying victimization, promote resilience, and contribute to high satisfaction rates with life by the students (Aldridge et al., 2020).

### Moral Disengagement and Bullying

Establishing the personal characteristics that define bullies is complex because some people who present these characteristics are frequently not bullies, whereas others who a priori do not have these characteristics end up bullying (Zych et al., 2017a). However, a multitude of research has shown the importance of the mechanisms of moral disengagement in the development of bullying behaviors.

Moral disengagement refers to “socio-cognitive maneuvers that allow people to disengage from moral rules without any sense of remorse, guilt, or self-condemnation” (Bandura, 1999, p. 194). In this way, moral disengagement allows young people to justify bullying perpetration, despite understanding that these behaviors are generally inappropriate. This mechanism allows transgressing the code of ethics itself to perform behaviors, in principle unacceptable, in certain situations without feeling guilty. Thus, the positive relationship between moral disengagement and bullying perpetration are well documented (e.g., Bjärehed et al., 2020; Gini et al., 2020; Travlos et al., 2021), as indicated by recently developed meta-analyses (Gini et al., 2014; Killer et al., 2019). They show how bullying perpetrators trigger different mechanisms of moral disengagement to avoid feelings of guilt or shame. Thus, adolescents can find moral justification for attacking someone if they consider that they are helping their friends, their attitudes are not so serious compared to others possible attitudes, their attitudes are only a joke, or they are not guilty by abusing someone if other people have mistreated them before. Sometimes students believe that classmates' differences justify the aggression (Gini et al., 2014; Killer et al., 2019).

The social environment can compel young people to manifest certain antisocial behaviors, in line with Chan and Wong (2015b, p. 105), who showed that “bullying behaviors, in Chinese societies, have been regarded as a collectivist conduct as a mean to maintain group conformity.” According to some authors (Montero-Carretero and Cervelló, 2019), developing a robust moral identity would help young people face social pressure situations and impose an ethical and moral code in which aggression has no place. Other authors showed the importance of promoting social consensus (Reynolds and Ceranic, 2007), understood as the degree to which a specific action is considered more or less acceptable by people who make up the environment. There is a higher prevalence of bullying in groups with a high collective moral disengagement level (Thornberg et al., 2021).

Although many studies showed moral disengagement as a predecessor of bullying, few works have used longitudinal designs to examine the causal effect of moral disengagement on bullying perpetration over time. Teng et al. (2020), in a study conducted with 2,997 Chinese adolescent students, with measures at three times, analyzed the association between moral disengagement and bullying perpetration, further exploring the moderating effect that the students' perceptions of school climate had on those relationships. Their results showed that (a) students with higher moral disengagement and more negative perceptions of school climate perpetrated more bullying than those with lower moral disengagement and more positive perceptions of school climate; (b) students with higher values of moral disengagement and negative perceptions of school climate presented higher levels of bullying perpetration over time; (c) the association between moral disengagement and bullying perpetration was weaker and nonsignificant for students with more positive perceptions of school climate. These findings encourage further research of the protective effect that a student's perception of a positive school climate can have on the relationship between moral disengagement and bullying perpetration.

### Empathy and Bullying

Empathy has been defined as a personality trait that grants the ability to perceive the moods of others and to become cognitively and affectively aware of them (Garaigordobil, 2009). The existence of two dimensions follows from this definition: cognitive empathy is defined as the ability to understand the emotions of others, whereas affective empathy refers to the ability to experience the emotions of others.

The lack of empathy of some young people would make it impossible for them to put themselves in the place of others, and understand and share their emotions, which could make it easier for them to become aggressors, as indicated by many previous studies on traditional bullying (e.g., Mitsopoulou and Giovazolias, 2015) and cyberbullying (Kowalski et al., 2014; Baldry et al., 2015). Other authors showed that the lack of empathy is a characteristic not only from aggressors but also from the victims (Chan and Wong, 2015a, 2020).

Del Rey et al. (2016) showed that low levels of affective and cognitive empathy predicted bullying perpetration and cyberbullying in different groups of age, gender, and nationalities (including Spain and Greece). The results of a recent meta-analysis (Zych et al., 2019) that analyzed the role of empathy among other personal antecedents of bullying reflected that both the cognitive and affective dimensions had negative relationships with bullying perpetration in many previous works. In the same meta-analysis, it was shown that “callous-unemotional,” a construct that defines the lack of empathy and remorse, has shown positive relationships with bullying perpetration in numerous studies. These data confirm the importance of empathy as a personal characteristic present in aggressors and justifies its inclusion in predictive models that try to analyze the relationships between variables to explain bullying perpetration.

### The Present Study

The main purpose of this study was to analyze the interactions between contextual and personal antecedents affecting bullying perpetration. School climate will be analyzed using the instrument recently created by Aldridge et al. (2016) to tap into the factors that correspond to an environment that promotes the prevention of aggressive behavior at school, in terms of the role of teachers and peers and the educational and organizational strategies.

Specifically, the study aims to contribute to the existing literature in two respects: (a) examining the moderating effect of the different dimensions of school climate on the experiences of victimization, moral disengagement, and empathy; (b) analyzing how victimization, moral disengagement, and empathy mediate the relationships between school climate and bullying perpetration.

**Figure 1:**
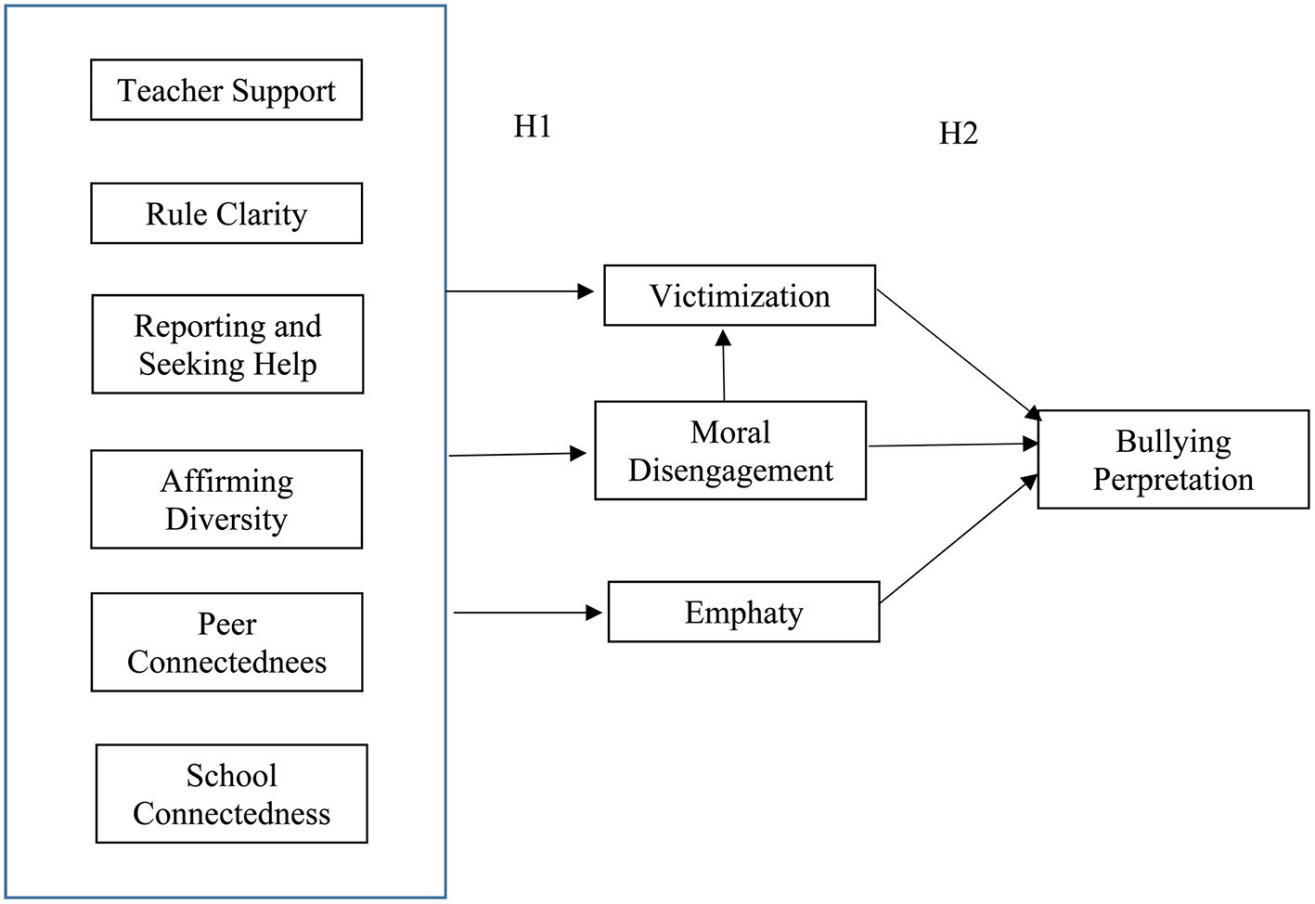
Hypothetical sequential model of the prediction of school climate, victimization, moral disengagement, empathy, and bullying perpetration.

Drawing on prior literature, the following specific hypotheses were formulated (Figure 1).

Hypothesis 1. We expect that the different dimensions of school climate will moderate victimization, moral disengagement, and empathy. Specifically, we expect that teacher support, rule clarity, and school connectedness will negatively predict victimization, whereas reporting and seeking help, and affirming diversity may positively predict it (Hypothesis 1a), as in the study of Aldridge et al. (2018). We also expect that teacher support, rule clarity, reporting and seeking help, affirming diversity, and peer connectedness will negatively predict moral disengagement (Hypothesis 1b). We do not know of any works that have analyzed these relationships previously, so we draw on the study of Montero-Carretero and Cervelló (2019), where these dimensions of school climate positively predicted moral identity, in addition to studies that have revealed positive school climates as predictors of positive emotional and behavioral consequences (Wang et al., 2010). Due to this principle, we expect that the different dimensions of school climate will positively predict empathy (Hypothesis 1c). Acosta et al. (2019) found positive relationships between school connectedness, peer connectedness, and empathy. They also reported that the higher the level of students' empathy, the greater the likelihood that they would report bullying, which is related to the school climate dimension of reporting and seeking help.

Hypothesis 2. We expect victimization, moral disengagement, and empathy will predict bullying perpetration, mediating the relationship between school climate and such perpetration. Specifically, we expect that victimization will positively predict bullying perpetration (Hypothesis 2a), based on the works that have revealed how aggressors were often victimized in the past (Cook et al., 2010; Hemphill et al., 2012; Zych et al., 2017b) and the authors who have shown the overlap between perpetration and victimization (Chan and Wong, 2015a, 2020). We also expect that moral disengagement will positively predict bullying perpetration and that empathy will negatively predict it (Hypothesis 2b), based on a multitude of works that have previously shown this outcome with moral disengagement (Gini et al., 2014; Killer et al., 2019; Teng et al., 2020) and empathy (Del Rey et al., 2016; Zych et al., 2019).

## Method

The project summary, hypotheses, and analysis plan have been registered with the Open Science Framework. A link to study registration materials can be found here: https://osf.io/scdhb/?view_only=caa459b1370d46798d258d8ec6d724b6.

### Participants

The sample was composed of 629 students (304 boys and 325 girls) between the ages of 12 and 14 (*M* = 12.55, *SD* = 0.67). They come from eight schools (five public and three concerted) from the province of Alicante (Spain) participated in the study. Regarding the grade, 173 studied 6th grade of Primary Education, 248 studied 1st grade of Compulsory Secondary Education (CSE), and 208 studied 2nd grade of CSE.

### Procedure

First, a random cluster sample was selected of the schools of Alicante (province of Spain). The school directors were then contacted to request them to participate and inform them about the objectives of the study, as well as its exclusively scientific and academic purposes. They were informed of the anonymous and voluntary nature of the test, as well as the strict confidentiality of the data obtained therein.

Once the school directors had agreed, a written statement was sent to request the informed consent of the parents and the Autonomous Secretariat of Education, which gave its authorization (file 05ED01Z/2017. 56.).

After obtaining the necessary permits and authorizations, the teachers in charge were coordinated on the day of the surveys. Data collection was carried out in a classroom of each school in one of the classes scheduled for physical education during the first trimester of the academic course 2017/2018. Before the test, students were instructed about the importance of being sincere in their responses. During the completion of the questionnaires, the doubts that arose were clarified by the teacher of the subject, who had previously been instructed by the researchers. The questionnaires were completed anonymously in ~ 20 min.

### Measuring Instruments

The measurement instruments are presented below, along with the internal consistency indices of each factor. For those instruments that have never been used in Spanish, a confirmatory factorial analysis is also presented.

#### Measurement of School Climate

An adaptation of the instrument designed by Aldridge et al. (2016) was used. The original instrument, called What Is Happening In This School? (WHITS), was validated in Spanish by Montero-Carretero and Cervelló (2019). This questionnaire captures factors that correspond to a favorable environment for the prevention of aggressive behavior at school, in terms of the role of teachers, peers, and the educational and organizational strategies of the school.

The introductory stem was, “In this school or institute…” grouping the answers into six dimensions of school climate. These dimensions were: (1) *Teacher Support*, with four items (e.g., “the teachers try to understand my problems”); (2) *Rule Clarity* with four items (e.g., “I understand why the school rules are the way they are”); (3) *Reporting and Seeking Help* with four items, (e.g., “I can report incidents without others knowing”); (4) *Affirming Diversity* with four items (e.g., “the days that are important to my culture are recognized”); (5) *Peer Connectedness* with three items (e.g., “the students support me”); (6) *School Connectedness* with four items (e.g., “I like being in school”).

Responses are formulated on a numeric Likert scale ranging from 1 (*almost never)* to 5 *(almost always*).

Regarding reliability, all the factors showed alphas above 0.70, except for the School Connectedness factor, which had alpha indices below 0.60, so we decided not to include it in the analysis.

#### Measurement of Moral Disengagement

The 18-item Moral Disengagement in Bullying Scale (MDBS) was used to measure the degree to which students morally disengage from bullying situations. This instrument was validated for schoolchildren by Thornberg and Jungert (2013). The instrument consists of a general factor of *Moral Disengagement*, based on the definition of moral disengagement of Bandura (1999). The scale also has seven first-order factors, which are *Moral Justification* (e.g., “it's okay to hurt a person a couple of times a week if you do it to help your friends”), *Euphemistic Labeling* (e.g., “saying bad things to a certain person a couple of times a week doesn't matter. It's just a little joke”), *Advantageous Comparison* (e.g., “making fun of a person a couple of times a week is no big deal, it's much worse to beat them up every week”), *Displacement of Responsibility* (e.g., “if students have parents who do bad things to them, it's not their fault if they then bully other students”), *Diffusion of Responsibility* (e.g., “a student can't avoid bullying another student if all his friends are doing it”), *Distorting Consequences* (e.g., “surely, it won't hurt you if they make fun of you from time to time.”), and *Victim Attribution* (e.g., “it's okay to intimidate those who aren't like everyone else”). Students graded each item on a seven-point scale, ranging from (*disagree*) to 7 (*agree*).

As there was no prior validation of the instrument, we subjected the Scale of Moral Disengagement in Bullying to a confirmatory factorial analysis with seven first-order factors and a second-order factor (*Moral Disengagement*), finding appropriate fit indices, [χ^2^ = 432.03; χ^2^/*df* = 3.37; CFI = 0.92, TLI = 0.90, SRMR = 0.051, RMSEA = 0.061 (95% CI (0.055, 0.068), *p* < 0.003].

The first-order scales' alpha coefficient ranged from.66 to.83, acceptable values keeping in mind that dimensions with values below 0.70 were composed of lesser than four items (Loewenthal, 2001). The second-order factor (*Moral Disengagement*) obtained an alpha of 0.88, showing a high level of internal consistency, so we decided to include the second-order factor in the analyses.

#### Measurement of Empathy

To measure empathy, the Spanish version (Villadangos et al., 2016) of the Basic Empathy Scale of Jolliffe and Farrington (2006) was used. This scale consists of two factors that measure the cognitive and affective empathy dimensions, composed of 20 items, where the answers are rated on a 5-point Likert scale ranging from 1 (*totally disagree*) to 5 (*totally agree*). Nine items measure *Cognitive Empathy* (e.g., “I understand the joy of my friends when something works out for them”) and 11 items measure *Affective Empathy* (e.g., “after being with a friend who is sad about something, I usually feel sad”). The answers are rated on a 5-point Likert scale ranging from 1 (totally disagree) to 5 (totally agree).

The alpha coefficients for the scales were 0.78 and 0.80 for *Cognitive* and *Affective Empathy*, respectively. The alpha coefficient was also calculated for the total of the empathy items, finding that *Global Empathy* had an alpha of 0.82.

#### Measurement of Bullying

The Spanish version of the European Bullying Intervention Project Questionnaire (EBIP-Q), of Ortega-Ruiz et al. (2016) was used to measure this variable.

This scale includes two factors, which reflect the behaviors of Bullying (*Victimization and Perpetration*) with seven items each. The first seven items are related to *Victimization*, describing situations such as: “Someone has stolen or broken my things,” “Someone has threatened me,” “Someone has insulted me.” The last seven items are related to *Perpetration*, describing situations such as: “I've stolen or ruined someone's things,” “I've threatened someone,” “I've spread rumors about someone.” Students are asked to indicate how often they have performed or suffered these behaviors in the past two months. Each item is formulated through direct questions in the first person. The student must answer them on a five-point Likert scale, as follows: 1 (*No*), 2 (*yes, once or twice*), 3 (*yes, once or twice a month*), 4 (*yes, about once a week*) to 5 (*yes, more than once a week*). The alpha coefficients were 0.82 for the *Victimization* and 0.78 for the *Perpetration*.

### Data Analysis

A descriptive and correlational analysis was carried out to explore the relationship of contextual (school climate) and personal variables (moral disengagement, empathy, victimization, and bullying perpetration). Moreover, as the work aimed to test whether the contextual and personal variables predicted bullying perpetration, a path-analysis was used to study the sequential model presented in the hypotheses (Figure 1).

To check the path-analysis fit, IBM SPSS Amos 19 software was used. The exploration of model fit indices followed the guidelines of Hu and Bentler (1999), considering a good fit index of the model chi-square/*df* values between two and three, with limits of up to five, incremental fit indices (CFI) and Tucker-Lewis fit indices (TLI) greater than 0.90, and error fit indices of less than 0.08 for the root mean square error of approximation (RMSEA), and 0.04 for the standardized root mean square residual (SRMR). Hu and Bentler (1999) recommend considering several of these indices to accept or reject a model, and not accept it with only one of these indexes or reject it for non-compliance with only one of the fit indices.

## Results

### Descriptive Statistics and Correlation Analysis

Table 1 shows the descriptive statistics and correlations between the variables of the study. The means of the factors showed moderate to high values of perception of school climate and empathy and lowers moral disengagement levels, taking into account the response range of these variables (1–5). Specifically, students perceived greater affirming diversity (*M* = 4.18, SD = 0.87), followed by peer connectedness (*M* = 3.86, SD = 0.85), reporting and seeking help (*M* = 3.77, SD = 0.96), rule clarity (*M* = 3.74, SD = 0.95), and teacher support (*M* = 3.42, SD = 1.06). The students showed higher levels of empathy (*M* = 3.52, SD = 0.57) than moral disengagement (*M* = 1.87, SD = 0.86). Bullying perpetration and victimization showed lower values, considering the response range of these variables (1–7). The means were higher for victimization (*M* = 1.55, SD = 0.67) than for bullying perpetration (*M* = 1.28, SD = 0.45). It can be seen that the correlations are in agreement with the proposed hypotheses.

**Table 1:** Descriptive statistics and correlations between the perception of the school climate, victimization, moral disengagement, empathy, and bullying perpetration.

	***M***	***SD***	**1**	**2**	**3**	**4**	**5**	**6**	**7**	**8**
1. Teacher support	3.42	1.06								
2. Rule clarity	3.74	0.95	0.49^**^							
3. Reporting and seeking help	3.77	0.96	0.37^**^	0.52^**^						
4. Affirming diversity	4.18	0.87	0.27^**^	0.44^**^	0.43^**^					
5. Peer connectedness	3.86	0.85	0.36^**^	0.40^**^	0.42^**^	0.45^**^				
6. Victimization	1.55	0.67	−0.15^**^	−0.16^**^	−0.16^**^	−0.10^**^	−0.12^**^			
7. Moral disengagement	1.87	0.86	−0.05	−0.10^*^	−0.11^**^	−0.12^**^	−0.15^**^	0.15^**^		
8. Empathy	3.52	0.57	0.16^**^	0.31^**^	0.30^**^	0.31^**^	0.34^**^	−0.00	−0.16^**^	
9. Bullying perpetration	1.28	0.45	−0.16^**^	−0.23^**^	−0.18^**^	−0.16^**^	−0.21^**^	0.51^**^	0.35^**^	−0.19^**^

* *p < 0.05;*

** *p < 0.01*.

Thus, we find direct and significant relationships between all the dimensions of school climate and empathy; Specifically, we find positive relationships between empathy and teacher support (*r* = 0.16, *p* < 0.01), rule clarity (*r* = 0.31, *p* < 0.01), reporting and seeking help (*r* = 0.30, *p* < 0.01), affirming diversity (*r* = 0.31, *p* < 0.01), and peer connectedness (*r* = 0.34, *p* < 0.01). Moral disengagement correlated significantly and negatively with the dimensions of school climate, except for teacher support. The significant correlations were (*r* = −0.10, *p* < 0.05) for rule clarity, (*r* = – 0.11, *p* < 0.01) for reporting and seeking help, (*r* = −0.12, *p* < 0.01) for affirming diversity, and (*r* = −0.15, *p* < 0.01) for peer connectedness.

Victimization also correlates negatively with all dimensions of perception of school climate. Specifically, we find correlations between victimization and teacher support (*r* = −0.15, *p* < 0.01), rule clarity (*r* = −0.16, *p* < 0.01), reporting and seeking help (*r* = −0.16, *p* < 0.01), affirming diversity (*r* = −0.10, *p* < 0.01), and peer connectedness (*r* = −0.12, *p* < 0.01). We also find direct relationships between victimization, bullying perpetration (*r* = 0.51, *p* < 0.01), and moral disengagement (*r* =0.15, *p* < 0.01). Finally, a negative and significant correlation between empathy, bullying perpetration (*r* = –.19, *p* < 0.01), and moral disengagement (*r* = – 0.16, *p* < 0.01), was observed.

### Path-Analysis

**Figure 2:**
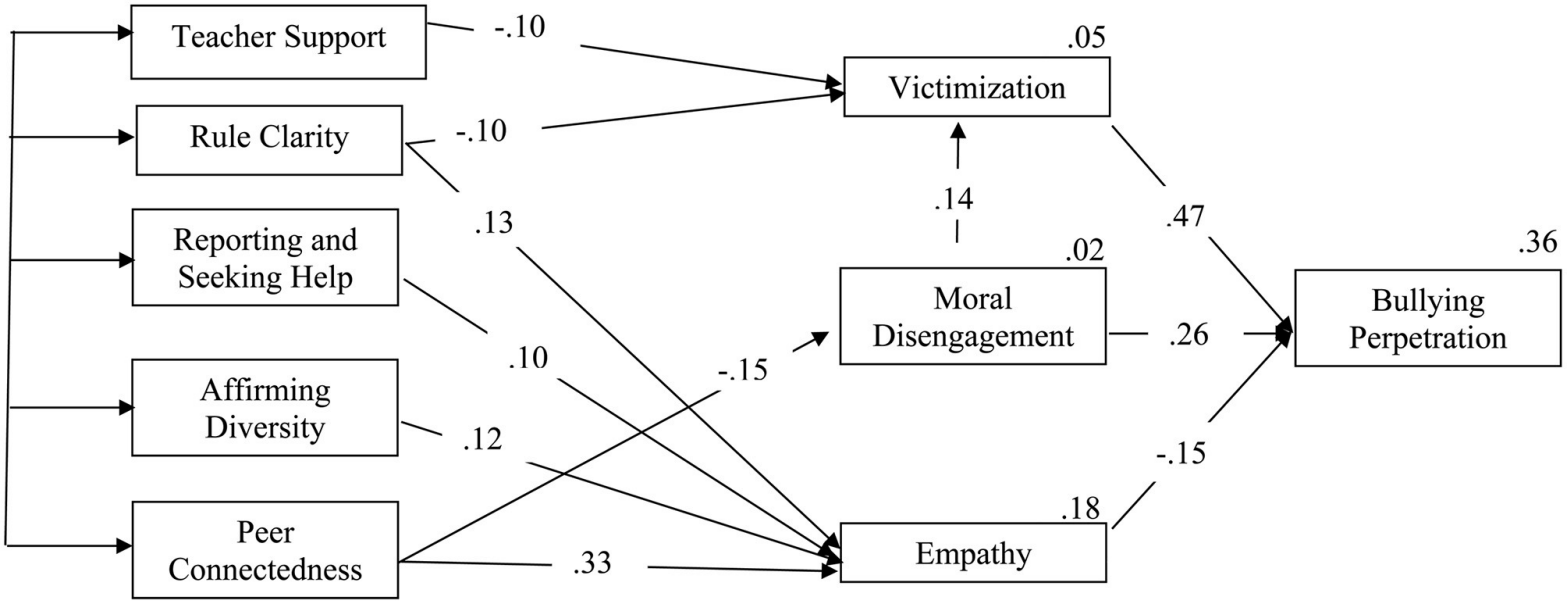
Standardized solution of the school climate, victimization, moral disengagement, empathy, and Bullying perpetration model. Only statistically significant paths are presented, and correlations between exogenous variables (values between 0.27 and 0.53, all significant, *p* < 0.05) were omitted.

To verify the sequential model proposed in the hypotheses, in which the perception of school climate would predict victimization, moral disengagement, and empathy, and these variables, in turn, would predict bullying perpetration, a path analysis was performed with IBM SPSS Amos 19 software, following the guidelines of Hu and Bentler (1999). Only those paths that showed significant predictions were included (Figure 2). The estimation method of the model was the maximum likelihood (ML), suitable for our model because the normal multivariate distribution was acceptable (Mardia coefficient = 31.19), taking into account that values < 70 indicate normality (Hu and Bentler, 1999).

The analysis showed good fit indices both for incremental fits and error indices [χ^2^ = 74.81; χ^2^/*df* = 3.74; CFI = 0.97, TLI = 0.94, SRMR = 0.04, RMSEA = 0.06, (95% CI (0.029, 0.08), *p* < 0.03]. The direct effects of school climate on the mediation variables showed that teacher support (β = −0.10, *p* < 0.05), and rule clarity (β = −0.10, *p* < 0.05) negatively predicted victimization. Peer connectedness, negatively (β = −0.15, *p* < 0.05) predicted moral disengagement. Rule clarity (β = 0.13, *p* < 0.05), reporting and seeking help (β = 0.10, *p* < 0.05), affirming diversity (β = 0.12, *p* < 0.05), and peer connectedness positively predicted empathy (β = 0.33, *p* < 0.01).

Path analysis also showed that moral disengagement, positively predicted victimization (β = 0.14, *p* < 0.05). Finally, bullying perpetration was positively predicted by victimization (β = 0.47, *p* < 0.01), and moral disengagement (β = 0.26, *p* < 0.01), and negatively by empathy (β = −0.15, *p* < 0.05). The indirect effects showed that all the factors of school climate negatively predicted bullying perpetration (values between −0.015 and −0.078).

## Discussion

This study complements the previous literature by examining the impact of school climate on victimization, moral disengagement, and empathy, as well as the mediating effect of these three variables on the relationships between school climate and bullying perpetration.

While school climate, victimization, moral disengagement, and empathy have already been analyzed in previous studies as predictors of bullying perpetration (Cook et al., 2010; Gini et al., 2014; Chan and Wong, 2015a; Konishi et al., 2017; Killer et al., 2019; Zych et al., 2019), this research differs from these by analyzing an unknown model, from a more complete conceptualization of psychosocial school climate along with all these antecedents.

The results of this work corroborate those of previous studies that have indicated that the interaction between contextual factors and personal characteristics determines the appearance of bullying perpetration (e.g., Chan and Wong, 2020).

The results will be discussed in relation to the two research hypotheses proposed, and some directions that future research could follow will be identified.

*Hypothesis 1*. The impact of school climate on victimization, moral disengagement, and empathy.

The results partially confirm Hypothesis 1a, as two of the six dimensions of school climate predicted victimization (see Figure 2). Both dimensions (teacher support and rule clarity) did so negatively, indicating that students' more positive perceptions of those aspects of school climate are associated with lower levels of victimization, as was the case in the work of Aldridge et al. (2018). In that study, school connectedness also negatively predicted victimization. In this study, the dimension known as school connectedness did not present acceptable reliability indices in the psychometric analysis, so it was not included in the model. This means that the results in this dimension could not be compared with those of Aldridge et al. (2018).

Teacher support has previously been linked to decreases in bullying (Olweus, 1994; Demaray and Malecki, 2003; Li et al., 2011), so our results reinforce the importance of teaching styles to prevent students from being victimized. In fact, in a recent study performed with Spanish students of the same ages as those of this work, teachers' autonomy support style, characterized among other things by being close and accessible to students and concerned about their problems, negatively predicted victimization mediated by resilience (Montero-Carretero and Cervelló, 2020).

In addition, considering that the field of moral development has moved toward an identity model based on the theory of social identity (Aquino and Reed, 2002), we expected that different dimensions of school climate would negatively predict moral disengagement (Hypothesis 1b). This hypothesis was partially met, as only peer connectedness did so. Montero-Carretero and Cervelló (2019) showed that five of the six dimensions of school climate predicted moral identity, in which peer connectedness was the strongest predictor, as in Aldridge et al. (2016) and Read et al. (2015). Relationships with peers are probably one of the most contextual aspects of young people's moral development and all the decision-making mechanisms in which they are judged socially. In this line, some intervention programs have been shown to be effective in preventing bullying through the promotion of adolescents' moral identity (Montero-Carretero et al., 2021).

Based on our results, the quality of peer relationships is a crucial aspect to ensure that students can prioritize their own code of ethics in the face of situations where the context could drive them to transgress it. Teachers should be able to establish dynamics in which students promote positive peer interactions when addressing the curriculum objectives of the different subjects, considering that peer connectedness is one of the most decisive aspects through which, over time, young people develop prosocial behaviors and move away from intimidating behaviors (Vagos and Carvalhais, 2020).

In favor of Hypothesis 1c, different dimensions of school climate positively predicted empathy. Peer connectedness was the strongest predictor, which, along with rule clarity, reporting and seeking help, and affirming diversity, behaved like a predictor. These results are in line with those found by Acosta et al. (2019) and indicate the importance of the promotion of a positive climate, going from strategies such as making the rules about bullying explicit, facilitating channels to ask for help, and fostering respect for diversity in the school, besides the aforementioned promotion of activities that increase the quality of relations between students. These aspects could contribute to forming more empathetic students, and have already been taken into account in most bullying prevention programs that have managed to reduce its prevalence, as shown by some systematic reviews (e.g., Ttofi and Farrington, 2011). The approximation of Ang (2015) shows that training in general empathy and modifying the normative beliefs about aggression in intervention programs achieve better results.

*Hypothesis 2*. The mediating effect of victimization, moral disengagement, and empathy in the relationships between school climate and bullying perpetration.

The results confirm the second hypothesis, as victimization and moral disengagement positively predicted bullying perpetration, whereas empathy predicted it negatively. The fact that victimization positively predicted bullying perpetration (Hypothesis 2a) confirms the importance that past experiences seem to have, in which students who were victimized end up becoming aggressors, as other authors have pointed out. Hemphill et al. (2012), in a study focusing on the predictors of bullying and cyberbullying performed with Australian students, reported that those who informed having suffered some relational aggression tended to perpetrate bullying or cyberbullying. For bullying alone, perpetrators showed that they had previously been involved in bullying (as victims or perpetrators), and they had family problems and problems at school. The literature has described the profile of students who are victims and bullies (Chan and Wong, 2015a), under the term bully-victims.

Cook et al. (2010) pointed out defiant and aggressive behaviors, as well as the bad influence of peers, as antecedents, whereas self-esteem and positive school climate (through feelings of belonging to the school, fair treatment, and respect) were identified as protective factors. It should be noted that this study produced an unexpected result, as moral disengagement positively predicted victimization. This result could be precisely because many of the participating students could respond to that profile of bully-victim. However, more research is needed to determine whether this result is a characteristic of our sample or whether a strong relationship appears in other works.

Our results also confirm Hypothesis 2b and are in line with those of many previous studies that have shown the predictive nature of moral disengagement and empathy on bullying perpetration (Killer et al., 2019; Zych et al., 2019). In line with our results, Kokkinos et al. (2016) pointed to moral disengagement as the main predictor of relational aggression. This study also showed the importance of the quality of peer relationships concerning moral disengagement and relational aggression.

Therefore, it seems highly recommendable for the educational system to be concerned with building social consensus within the school about bullying behaviors, where aggression is never justified, in order to make it difficult for students to develop mechanisms of moral disengagement. Considering the results of Hein et al. (2015), teachers should undertake that task by avoiding controlling styles, which could provoke effects contrary to the desired ones, through anger in students.

It also seems advisable to help students identify the emotions that generate some stressful situations for peers, training them to put themselves in each other's place under an empathetic perspective that helps them adopt behaviors other than bullying. The design of school climates based on the promotion of the dimensions measured in this study could be of great use for this purpose. Casas et al. (2013) have already shown the relationships that are established between some dimensions of school climate and empathy as antecedents of bullying perpetration in Spanish high school students. In this study, teacher support was instrumental in improving peer relationships and promoting empathy in a work where both variables negatively predicted bullying perpetration.

Our results confirm those of Acosta et al. (2019), demonstrating the important role that the perception of a positive school climate can play as a moderator of the personal characteristics with bullying perpetration. As some authors have suggested (e.g., Chan and Wong, 2015b), it should be recommendable to establish prevention programs from a whole-school approach to generate a positive school climate.

## Implications of The Findings

The results of the present study implicate some practical consequences. On the one hand, school management teams should implement operating policies for the entire school concerning bullying. These policies should involve teachers, students, families, and staff, with a public regulation on bullying. Students must perceive that diversity is accepted and understood as a positive value at school.

Besides, it seems appropriate for teachers to be near the students, offering support and enabling channels to communicate their problems. Dynamics that encourage peer relationships are recommended, such as cooperative work where students try to achieve common goals or exercises in which students have the opportunity to communicate effectively with their peers about their emotions.

Considering that the lack of empathy is one of the main precursors of bullying, teachers could guide students' dynamics to identify their classmates' emotions. Different teachers should make curricular adaptations that allow the generation of social consensus, where the different forms of bullying cannot be justified, avoiding moral disengagement mechanisms.

It seems essential that schools activate protocols for observing the behaviors of victims and work with them to help them avoid becoming future bullies.

## Limitations and Lines of Future Research

This study has some limitations. Comparing our study with others that have approached the topic from correlational methodologies, it would be convenient for future research to study a more significant number of participants, with a higher age range, with populations of different socioeconomic levels. It would also be interesting to replicate the present study in special populations, such as people with disabilities.

New works should be carried out with both longitudinal and experimental designs, which study in greater depth the modification of school climates, developing specific programs that influence both the improvement of empathy and the decrease of moral disengagement, because of the great relevance they have shown in this study for the prediction of bullying.

On another hand, we have also warned that one dimension of the measuring instruments used have reliability problems. Specifically, the school connectedness dimension was eliminated from the school climate because it did not reach the minimum required reliability indices. Future research should analyze if this is a characteristic of our study or a problem inherent in defining the factor. Although the instrument used in this study to measure school climate has shown good psychometric properties in studies carried out with Australian students, we only know of one previous work that used this scale with Spanish students (Montero-Carretero and Cervelló, 2019), and the School Connectedness factor also showed low-reliability values. A recent review study that analyzed the instruments to measure this factor showed the difficulties of unifying criteria concerning its definition and suggested future research to improve this measurement (García-Moya et al., 2019).

## Conclusions

The results of our study are in line with the social-ecological theory and the numerous authors who have pointed out that bullying is the product of an interaction between individual characteristics and different layers of social contexts. This work increases knowledge about how school climate can moderate moral disengagement and empathy, determinants in the development of bullying perpetration, and it highlights the importance of the figure of the bully-victim. Our results could contribute to the development of policies based on the development of positive climates in schools interested in preventing bullying among their students, through the improvement of the variables that have been shown to be antecedents.

## Data Availability Statement

The datasets presented in this article are not readily available because participants only authorized the researchers to use their data in this project. Requests to access the datasets should be directed to Carlos Montero (cmontero@umh.es).

## Ethics Statement

The studies involving human participants were reviewed and approved by Autonomous Secretariat of Education (code 05ED01Z/2017. 56.). Written informed consent to participate in this study was provided by the participants' legal guardian/next of kin.

## Author Contributions

All authors participated in the conceived and designed analysis. CM-C collected data. CM-C, EC, and FS-R performed analysis. All authors participated in writing the paper. All authors contributed to the article and approved the submitted version.

## Conflict of Interest

The authors declare that the research was conducted in the absence of any commercial or financial relationships that could be construed as a potential conflict of interest.
